# A Priority-Based Adaptive MAC Protocol for Wireless Body Area Networks

**DOI:** 10.3390/s16030401

**Published:** 2016-03-18

**Authors:** Sabin Bhandari, Sangman Moh

**Affiliations:** Department of Computer Engineering, Chosun University, 309 Pilmun-daero, Dong-gu, Gwangju 61452, Korea; sabinbhd@gmail.com

**Keywords:** wireless body area networks, mobile healthcare, medium access control, quality of service, traffic priority, coexistence, unlicensed band

## Abstract

In wireless body area networks (WBANs), various sensors and actuators are placed on/inside the human body and connected wirelessly. WBANs have specific requirements for healthcare and medical applications, hence, standard protocols like the IEEE 802.15.4 cannot fulfill all the requirements. Consequently, many medium access control (MAC) protocols, mostly derived from the IEEE 802.15.4 superframe structure, have been studied. Nevertheless, they do not support a differentiated quality of service (QoS) for the various forms of traffic coexisting in a WBAN. In particular, a QoS-aware MAC protocol is essential for WBANs operating in the unlicensed Industrial, Scientific, and Medical (ISM) bands, because different wireless services like Bluetooth, WiFi, and Zigbee may coexist there and cause severe interference. In this paper, we propose a priority-based adaptive MAC (PA-MAC) protocol for WBANs in unlicensed bands, which allocates time slots dynamically, based on the traffic priority. Further, multiple channels are effectively utilized to reduce access delays in a WBAN, in the presence of coexisting systems. Our performance evaluation results show that the proposed PA-MAC outperforms the IEEE 802.15.4 MAC and the conventional priority-based MAC in terms of the average transmission time, throughput, energy consumption, and data collision ratio.

## 1. Introduction

With rapid advancements in physiological sensors and wireless communication, wireless sensor networks have grown significantly, supporting a wide range of applications including healthcare and medical services. A wireless body area network (WBAN) is a special-purpose sensor network designed to connect various sensors and actuators located on/inside the human body for continuous monitoring of vital signs like heart rate, temperature, blood pressure, electrocardiograms (ECGs), electroencephalography (EEG), *etc.* [1].

Quality of service (QoS), flexibility, and cost effectiveness are important goals to be achieved for healthcare and medical applications in WBANs. Different sensors placed in different parts of the human body, collect critical and non-critical information, and send them to the coordinator. Moreover, different actuators can be placed within the vicinity, on/inside the human body to communicate with the coordinator. The inside or vicinity of a human body is a challenging environment for the design of adaptable, dynamic, and flexible protocols for WBANs. Therefore, in WBANs, low delay, high reliability, low power consumption, negligible electromagnetic interface with the human body, and effective communication are to be taken into consideration.

In general, MAC protocols play a crucial role in providing QoS and in prolonging network lifetimes by controlling packet collisions, overhearing, control overheads, and idle listening [2,3]. The IEEE 802.15.4 standard [4] exhibits desirable features for WBANs and has been applied to different WBAN platforms. However, there are several limitations for meeting specific requirements and considerations, for a successful implementation. In recent years, there have been several significant developments in MAC protocols for WBANs. A number of MAC protocols have been studied for specific purposes, but have been adopted with certain modifications to fulfill the inherent requirements of the WBANs. The IEEE 802.15.6 standard [5] defines the physical (PHY) and MAC layers to provide various services for healthcare and medical applications as well as other non-medical applications. The MAC layer in the IEEE 802.15.6 standard aims to support a low-complexity, low-cost, ultra-low power, and a highly reliable wireless communication.

In WBANs, MAC protocols have a great impact on the energy efficiency, reliability of communication, interference, and the QoS provision. In [6], different design approaches for the PHY and MAC layers, for efficient and reliable mobile healthcare services in WBANs, are discussed. In [7,8], various issues concerning channel modeling, coexistence, energy consumption, MAC layer issues, and design features are analyzed and summarized. More recently, MAC protocols for cognitive radio body area networks have been developed [9]. The MAC protocols developed so far for WBANs will be overviewed in Section 2.

In this paper, we propose a priority-based adaptive MAC (PA-MAC) protocol for WBANs in unlicensed bands. The beacon channel (BC) is used for the transmission and reception of beacon frames, while the data channel (DC) is used for the rest of the communication, unlike in IEEE 802.15.4. A fixed dedicated channel is assigned for a beacon. We prioritize the data traffic by using a priority-guaranteed carrier-sense multiple access with collision avoidance (CSMA/CA) procedure in the contention access period (CAP). To support the various QoS requirements, we classify the data traffic into four categories with different priorities and divide the CAP into four sub-phases dynamically, according to the number of nodes in each traffic category. The PA-MAC allocates time slots dynamically, based on the traffic priority. The proposed PA-MAC supports both the CAP and the contention free period (CFP). The CFP is used to transfer significant numbers of consecutive data packets to the coordinator. Further, multiple channels are effectively utilized to reduce the access delay in a WBAN, in the presence of coexistent systems. According to our simulation results, the proposed PA-MAC outperforms the IEEE 802.15.4 MAC and the conventional priority-based MAC in terms of the average transmission time, throughput, energy consumption, and the data collision ratio.

The rest of this paper is organized as follows: in the following section, related works are reviewed and discussed briefly. In Section 3, the principles and operations of the proposed PA-MAC protocol are presented in detail. In Section 4, the analytical approximation of the PA-MAC is described and discussed. In Section 5, the performance of the proposed PA-MAC is evaluated via computer simulation and compared with the IEEE 802.15.4 standard protocol and the conventional priority-based MAC protocol. Finally, this paper is concluded in Section 6.

## 2. Related Works

**Figure 1:**
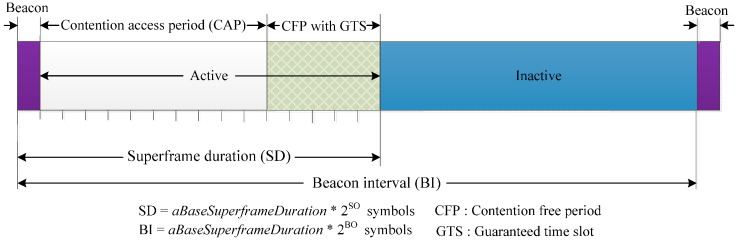
IEEE 802.15.4 superframe.

The IEEE 802.15.4 MAC protocol [4] operates in three frequency bands: 16 channels in the 2.4 GHz Industrial, Scientific, and Medical (ISM) band, 10 channels in the 915 MHz ISM band, and 1 channel in the European 868 MHz band. In the IEEE 802.15.4, two operational modes are defined: the beacon enabled mode and the non-beacon enabled mode. In the beacon-enabled mode, the communication is synchronized and controlled by the network coordinator. A superframe consists of active and inactive periods; the active period is further divided into three parts consisting of a beacon, a CAP using slotted CSMA/CA, and a CFP, as shown in Figure 1. The CFP contains up to seven guaranteed timeslots (GTS). All communication must take place during the active parts and the devices can sleep in an inactive part to conserve energy. The structure of the superframe is determined by the network coordinator using two parameters, the superframe order (SO) and the beacon order (BO). The SO is used to describe the length of superframe duration (SD), whereas, the BO defines the beacon interval (BI). There are mainly two types of devices in the IEEE 802.15.4: a full function device (FFD) and a reduced function device (RFD). The FFD can support all the network functions and operate as the network coordinator as well as an end device, whereas, the RFD can only be used as an end device. The FFD performs energy detection (ED) to detect the peak energy of a channel and select the appropriate channel for data transmission.

**Figure 2:**
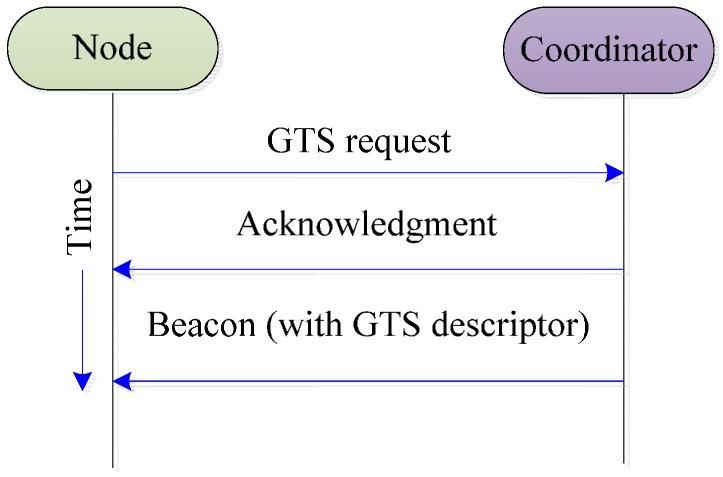
GTS allocation in IEEE 802.15.4.

In IEEE 802.15.4, if a node wants to reserve the resources for periodic traffic, it should first send a GTS request during the CAP with a CSMA/CA and the network coordinator will decide the GTS allocation accordingly. The GTS allocation scheme in IEEE 802.15.4 is shown in Figure 2. The working channel is statically selected by the network coordinator during the network initialization process. A WBAN working in an unlicensed band must share the medium with a set of coexisting systems like the Wi-Fi, Bluetooth, and the Zigbee that might cause beacon corruption and real-time connectivity problems. Moreover, the IEEE 802.15.4 MAC does not have any mechanism for data prioritization and all data traffic are treated with the same priority in the superframe.

The channel scheduling presented in [10] reduces the mutual interference between nodes belonging to the same network. To reduce idle listening, the control channel is differentiated from the data channels by using different frequency bands. The channel information is announced using beacon frames that are broadcast so that all the devices are aware of the assigned channels. However, this scheme does not consider priority differentiation mechanisms.

A priority-based channel access algorithm for a contention-based MAC protocol [11] is devised to solve the contention complexity problems. The algorithm categorizes traffic packets into four different levels and divides the CAP into four sub-phases, dynamically. In this algorithm, however, the classification of continuous and discontinuous data traffic and the use of GTSs are not considered.

A traffic-aware dynamic MAC protocol (TAD-MAC) for both invasive and non-invasive WBANs is introduced in [12]. In this protocol, each node adapts its wakeup interval dynamically, based on a traffic status register bank. The dynamic wakeup interval scheme saves the extra power consumed by idle listening, overhearing, collisions, and unnecessary beacon retransmissions.

The low-delay traffic-adaptive MAC protocol (LDTA-MAC) is reported in [13], where GTS time slots are allocated dynamically, based on node traffic to overcome the shortcomings of the IEEE 802.15.4 MAC protocol. Similarly, the schemes in [14] prioritize the data traffic based on the data features and adaptively allocate the CAP or CFP for the data according to the priority level. However, their traffic priority and backoff value are not considered.

In [15], a traffic load-aware sensor MAC (named ATLAS) is presented for collaborative body area sensor networks. The superframe structure dynamically varies based on the traffic load and uses a multihop communication pattern. Nevertheless, the priority of the different packets and the back-off classes are not considered.

A traffic priority and load-adaptive MAC (PLA-MAC) [16] provides QoS to the packets according to their traffic priority level. Packets with a higher priority level get better service than the packets with a lower priority. Although packet-level priority and reliability are considered, the channel adaptation of a condition-based network is not performed.

The traffic-adaptive MAC protocol described in [17] uses a traffic-based wakeup mechanism and a wakeup radio mechanism, to reliably accommodate various types of data. It utilizes the traffic information to enable low-power communication. Wakeup tables are established to coordinate the transmission schedules of the nodes, while a wakeup radio mechanism is employed for emergencies.

A schedule-based heartbeat driven MAC protocol (H-MAC) [18] uses the heart rhythm information to perform synchronization and reduces extra energy costs; however, the heart beat information is not always valid owing to variations in the patient’s health condition.

A context-aware MAC protocol [19] can switch between the normal state and the emergency state. The data rates and duty cycles of the sensor nodes are dynamically changed to meet the requirements of latency and traffic loads, in a context-aware manner. The sensor nodes can obtain one or more time slots for periodic or bursty applications, according to their traffic characteristics.

A hybrid and secure priority-guaranteed MAC protocol (PMAC) [20] uses two CAPs for accommodating normal and critical data, while one CFP is used for accommodating significant quantities of data packets. In addition, a set of security keys is used to prevent illegal access to the WBANs.

A MAC protocol specially designed for energy-harvesting WBANs is presented in [21]. The nodes are assigned different priorities and access methods, based on the criticality of their data packets and the type of the energy-harvesting source.

In [22], the robustness of the medical data packet transmission is experimentally investigated, based on the frequency hopping mechanism in heterogeneous environments. The measurement results demonstrate that the transmission reliability requirement depends significantly on the signal strength of the other signals as well as that of the chosen channel/frequency band. It is a fact that the heterogeneous working requirements of a WBAN, define different QoS issues that are specific to that particular application area only. WBAN applications are very sensitive; hence, QoS issues in WBANs require more attention and focus and are to be seriously considered.

## 3. Priority-Based Adaptive MAC (PA-MAC)

In this section, the proposed PA-MAC is presented in detail. Multiple channel utilization, data traffic prioritization, dynamic time slot allocation, and data transfer procedures are discussed.

### 3.1. Multiple Channel Utilization

**Figure 3:**
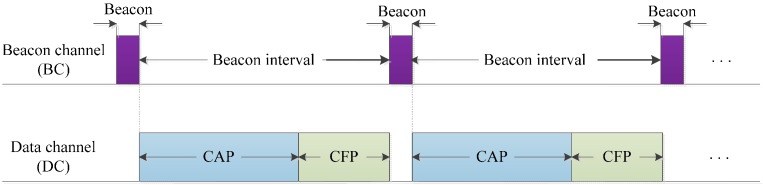
Channel switching mechanism.

**Figure 4:**
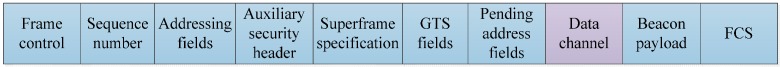
Data channel field in the IEEE 802.15.4 beacon frame.

In the proposed QoS-aware adaptive MAC, we exploit the channel switching capability of the IEEE 802.15.4 MAC radio hardware. Therefore, we implement two different channels: a dedicated beacon channel (BC) and a data channel (DC). The dedicated BC is available for exchange of control information like channel assignment broadcasts and access requests between the coordinator and the sensor nodes. The dedicated BC is used during the beacon frame transmission, whereas, the rest of the communication is done through the DC. During the beacon period, the node switches its channel to the BC and returns to its original DC at the end of the beacon period, as shown in Figure 3. The widely used transceivers for short range and low-power WPANs, e.g., the CC2420 and the more advanced CC2500, have a channel switching time of only 300 and 90 μs, respectively [23]. The DC information is conveyed to the sensor nodes by piggybacking the channel information on the beacon payload of the beacon frame, as shown in Figure 4. The entire network information can be determined just by scanning the BC [24]. In the 2.4 GHz ISM band, interference from high-power WLAN transmissions is dominant. Channels 13 and 14 of the IEEE 802.11 operating in the 2.4 GHz ISM band are not used by most of the WLAN systems in North America. Therefore, channel 25 or 26 of IEEE 802.15.4 WPAN would be free from WLAN interference and can be used as the dedicated BC. Although this scheme protects the beacon from WLAN interference, interference from IEEE 802.15.1 or other IEEE 802.15.4 WPANs may still exist. However, as the WPANs are generally operated at a lower transmission power, interference with these systems is negligible [25]. Channel selection schemes are not within the scope of this paper. The coordinator continuously senses all the channels in the pool of candidate channels; it assigns white spaces as the transmission slots to the body nodes. The coordinator may choose and remain tuned to an idle channel until it becomes unavailable or is degraded by the activities of the coexisting systems.

### 3.2. Data Traffic Prioritization and Dynamic Timeslot Allocation

Medical and non-medical applications are the two major categories in WBANs. Medical applications include healthcare and diagnosis-assistance related signal monitoring, whereas, non-medical applications cover signals related to consumer electronics (CE). In medical applications, emergency vital signals are directly related to the life of the patient, therefore, they should be regarded as first priority service. The priority levels for different kinds of data traffic are shown in Table 1.

**Figure 5:**
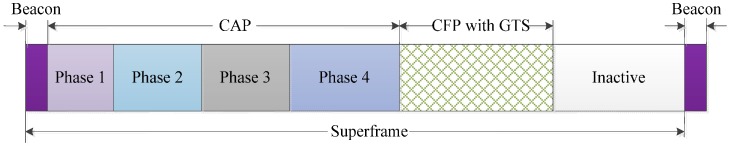
Superframe structure of the proposed MAC.

In the IEEE 802.15.4 MAC, the performance of the CAP significantly influences the collision probability and the final throughput. If the nodes are densely deployed in a narrow region, the contention complexities increase and lead to high collision ratios and significant energy consumption. The main goal of the proposed MAC is to provide QoS and low power consumption for various applications by dispersion of the contention complexity. Here, we divide the CAP into four sub-phases for each priority level of the traffic, as shown in Figure 5. Each specified access phase has dynamically changing length and is calculated by the proposed algorithm at the central coordinator.

On the other hand, the pure segregation of sub-phases for only one type of traffic in each in the CAP leads to wasted time slots. The other drawback of the pure segregation is low scalability with the traffic load; *i.e.*, the delay and data collision ratio also increase if the allocated time slot is not enough to handle offered traffic in the specific priority. When the offered traffic increases due to a large number of sensor nodes in the specific time slot, the collision probability increases significantly.

**Figure 6:**
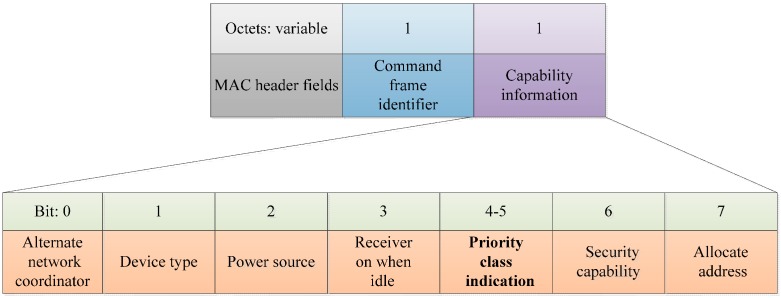
Association request command.

Traffic with priority *P*_1_ can access the channel in all phases; a node that transmits traffic with a priority *P*_4_, can use only phase 4. The *P*_2_ priority traffic can access the channel in phases 2–4. Similarly, *P*_3_ can access the channel in Phases 3 and 4. In order to avoid wastage of the timeslots, the lengths of the sub-phases are calculated dynamically, using Equation (1) [11]:

(1) $$l_{i}=\overset{i-1}{\underset{k=0}{\sum }}l_{k}+L_{CAP}*\left(N_{i}/N_{T}\right)$$

where $l_{i}$ is the length of the sub-phase i , (i=1,2,3,4) is taken from the starting point of CAP, $L_{CAP}$ is the length of the CAP, $N_{i}$ is the total number of nodes in the traffic category of priority $P_{i}$, $N_{T}$ is the total number of nodes, and $l_{0}\;$ is initially set to zero . To obtain information regarding the node’s priority classes, we modify the IEEE 802.15.4 association request command, as shown in Figure 6. Further, we assume that each node supports only one type of data. When newly arrived nodes join the network, the coordinator has the ability to sense the changes in the number of nodes of each class in the CAP of the previous superframe and calculate the value of the number of nodes in each traffic category. Medical services must satisfy a delay of 125 ms or less and consumer electronics (CE) services have to satisfy a delay requirement of 250 ms or less. Based on these delay requirements, the average transmission delay of each category is calculated by Equation (2):

(2) $$D_{k}^{i}=\alpha *D_{k-1}^{i}+\left(1-\alpha \right)*d_{k}^{i}$$

where $d_{k\;}^{i}$ is the delay of the *k-*th packet in the traffic category of priority $P_{i}$ and α = 0.125. If the delay threshold is exceeded, the CAP is divided into “the number of exceeded categories + 1” sub-phases.

### 3.3. Data Transfer Procedure

**Figure 7:**
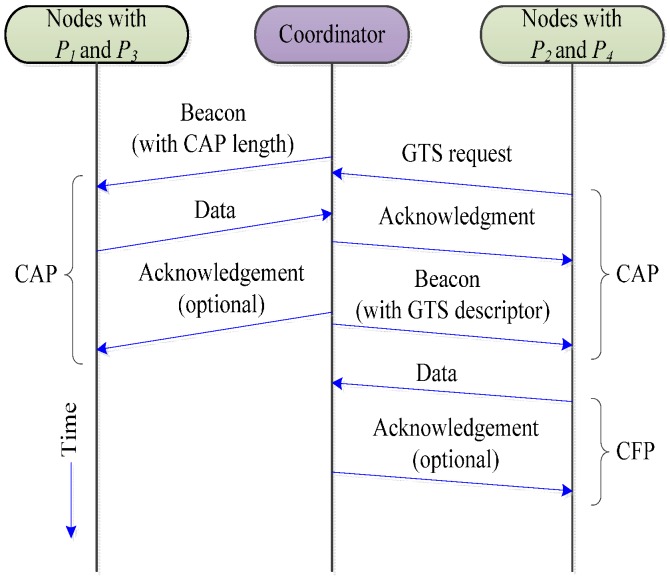
Data transfer procedure.

In the IEEE 802.15.4 MAC, the superframe consists of a CAP and a CFP. The CAP is suitable for the transfer of the command messages and short data, whereas, the CFP is implemented for continuous data. In the CAP, each node transmits its packets to a coordinator using the CSMA/CA procedure. In the CFP, each node transmits its packets to the coordinator by using dedicated guaranteed time slots (GTSs) without contention with the other nodes. In order to transmit packets in the CFP, each node transmits the request packet for the CFP to the coordinator in the CAP using the CSMA/CA procedure. When the coordinator successfully receives the GTS request packet, it allocates the GTS to the node, accordingly. The data from the *P*_1_ and *P*_3_ nodes are transmitted immediately after accessing the channel in the CAP. However, the *P*_2_ and *P*_4_ nodes send the GTS request command in the CAP to apply for GTS allocation. The slot allocation mechanism and data transfer procedure for different traffic priorities are shown in Algorithm 1 and Figure 7, respectively.

**Algorithm 1.** Algorithm for the CAP allocation**while** (!End of CAP)
1.1**if** (An associate request command packet is received from a node)1.1.1Calculate the number of nodes with different traffic priorities as *N_i_* ← *N_i_ +* 1, where *N_i_* is the total number of nodes in the traffic category of priority *P_i_* and i is an integer that varies from 1 to 4. 
 **end if****end while**2.Calculate the lengths of the sub-phases as $l_{i}=\sum_{k=0}^{i-1}l_{k}+L_{CAP}*\left(N_{i}/N_{T}\right)$, where $l_{i}$ is the length of sub-phase i from the starting point of the CAP, $L_{CAP}$ is the length of the CAP, $N_{T}$ is the total number of nodes, and $l_{0}$ is initially set to zero3.Broadcast the beacon frame

## 4. Analytical Approximation of the PA-MAC

In this section, we present the analytical approximation of the channel status, energy consumption, and the average delay of the proposed PA-MAC.

### 4.1. Channel Status

The low power and the low transmission rate of the WBAN nodes do not change the access pattern of the coexisting systems contending on a shared ISM band. The channels in the ISM band alternate between a busy state (occupied by a coexistent network) and an idle state when no coexistent system is accessing the channel. The channel state can be characterized by a two-state Markov chain. The average length of idle and busy periods depends upon the channel usage patterns of the coexisting systems. The length of the busy and idle periods for *j*-th licensed channel follows an exponential distribution with a mean busy time $\lambda_{j}$ and mean idle time $\;{µ}_{j}\;$[26]. The probability that the channel *j* is busy or idle at any time instant is given by:

(3) $$P_{j\left(busy\right)}=\frac{\lambda_{j}}{\lambda_{j}+{µ}_{j}}\text{ and }P_{j\left(idle\right)}=\frac{{µ}_{j}\;}{\lambda_{j}+{µ}_{j}\;}$$

The WBAN can access the medium as long as one of the *n* candidate channels is idle, and it loses its access when all the channels become busy owing to the activities of the coexisting systems. In the inactive state, all the WBAN operations and services are interrupted because there is no channel available for data transmission. The inactive state occurs with the probability:

(4) $$P_{inactive}=\;\overset{n}{\underset{j=1}{\prod }}P_{j\left(busy\right)}$$

When at least one channel becomes idle, the WBAN transits to the active state and its services are resumed. The probability of at least one channel becoming idle is calculated by:

(5) $$P_{active}=\;1-\overset{n}{\underset{j=1}{\prod }}P_{j\left(busy\right)}$$

### 4.2. Energy Consumption

**Figure 8:**
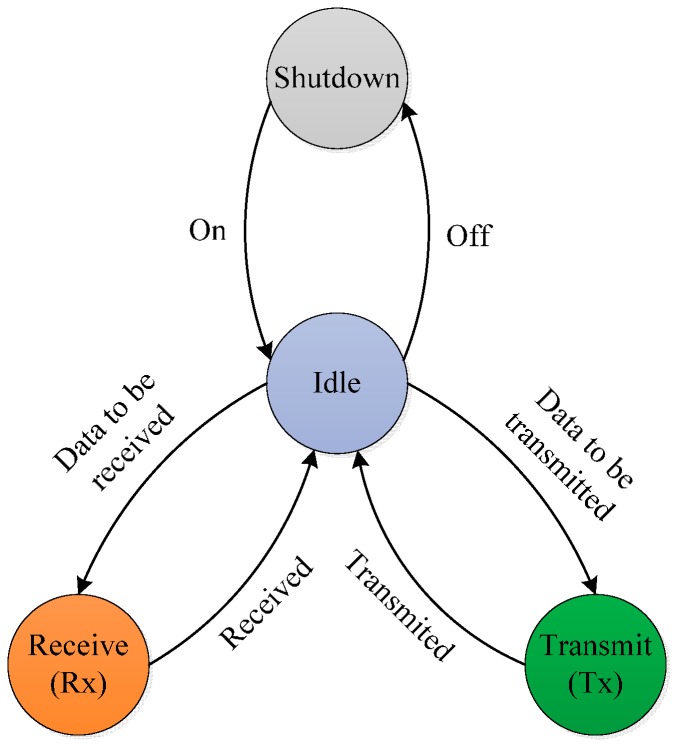
State transition of the transceiver.

Energy efficiency is one of the key measurement parameters for a reliable and efficient MAC protocol design. The energy consumption is based on the transceiver’s activity; the transition state of the transceiver is shown in Figure 8. To minimize the energy consumption, the idle and wakeup states play a vital role. We assume constant energy consumption by the sensing and processing units. Let $\;E_{C}$ be the total consumed energy in one cycle,$\;E_{I}$ is the energy consumed in an idle state, and $\;E_{W}$ is the energy consumed in a wakeup state. Then:

(6) $$\;E_{C}=E_{I}+\;E_{W}$$

The average total energy consumption for C number of cycles is given by:

(7) $$E_{T\left(avg\right)}=\overset{C}{\underset{c=1}{\sum }}\;E_{c}$$

Energy is a function of time and power and power itself is a function of voltage and current. In an idle state, the nodes consume less energy compared to the wakeup state. Therefore:

(8) $$\;E_{I}=T_{I}*\;P_{I}=\;T_{I}*\;I_{I}*V$$

(9) $$\;T_{I}=T_{F}-\;T_{W}$$

where $T_{F}$ is the total time-frame duration, $T_{I}$ is an idle time duration, $\;P_{I}$ is the power consumed in an idle state, and $\;I_{I}$ is the current drawn in an idle state from the voltage source, V.

In the wakeup time duration $\;T_{W}$, the nodes consume a switching energy $\;E_{SW}$, a transmission energy $\;E_{TX}$, and a reception energy $\;E_{RX}.$ Therefore:

(10) $$\;E_{W}=2\times \;E_{SW}+\;E_{TX}+\;E_{RX}$$

To switch between the ideal and wakeup state, the transceiver consumes an energy $\;E_{SW}$:

(11) $$\;E_{SW}=T_{SW}\times \;P_{SW}=\;T_{SW}\times \;I_{SW}\times V$$

where the node draws a current $\;I_{SW}$ from a voltage source during switching time duration $\;T_{SW}$ and $\;P_{SW}$ is the switching power. Let L be the length of the packet (control or data), $\;T_{TX}$ be the time needed for a single byte transmission, and $\;I_{TX}$ be the amount of current drawn during the packet transmission. Energy consumed during the transmission is given by:

(12) $$\;E_{TX}=L\;\times T_{TX}\times \;P_{TX}=\;L\;\times T_{TX}\times I_{TX\;}\times V$$

Similarly, the energy consumed at the receiver end is calculated as:

(13) $$\;E_{RX}=L\;\times T_{RX}\times \;P_{RX}=L\;\times \;T_{RX}\times \;I_{RX}\times V$$

where $\;P_{TX}$ and $\;P_{RX}$ are the power consumptions during the transmission and reception of the packets, respectively, $\;T_{RX}$ is the time needed for a single byte reception, and $\;I_{RX}$ is the amount of current drawn during the packet reception. Hence, the total average energy consumed is given by:

(14) $$E_{T\left(avg\right)}=\overset{C}{\underset{c=1}{\sum }}(T_{I}\times \;P_{I}+2\;\times T_{SW}\times \;P_{SW}+L\;\times T_{TX}\times \;P_{TX}+L\;\times T_{RX}\times \;P_{RX})$$

### 4.3. Transmission Time

**Figure 9:**
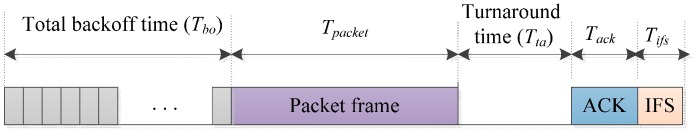
IEEE 802.15.4 frame transmission sequence.

The data frame transmission sequence is shown in Figure 9. $T_{bo}$ is the total backoff time (*i.e.*, channel access delay), $T_{packet}$ is the data packet transmission time, $T_{ta}$ is the transceiver’s turnaround time, $T_{ack}$ is an ACK frame transmission time, and $T_{ifs}$ is the time for an interframe space (IFS). The IFS could be either short inter-frame spacing (SIFS) or long inter-frame spacing (LIFS), depending upon the size of the MAC frame. The average transmission delay *T_l_* is the time needed to transmit a packet from the node to the coordinator and can be calculated as in [27] as follows:

(15) $$T_{l}=T_{bo}+T_{packet}+T_{ta}+T_{ack}+T_{ifs}$$

For *K* number of maximum back-off periods, the probability that the node can successfully access the channel is given by:

(16) $$P_{s}=\overset{K}{\underset{b=1}{\sum }}P_{a}{\left(1-P_{a}\right)}^{\left(b-1\right)}$$

where $P_{a}$ is the probability that a node can access the idle channel at the end of a backoff period. For *m* number of nodes in the network, $P_{a}$ is given by:

(17) $$\;P_{a}={\left(1-q\right)}^{\left(m-1\right)}$$

where q is the probability that a network device is transmitting at any time. The average number of back-off periods, R, is calculated as in [27] as follows:

(18) $$=\left(1-P_{s}\right)K+\overset{K}{\underset{b=1}{\sum }}bP_{a}{\left(1-P_{a}\right)}^{\left(b-1\right)}$$

The packet transmission time $T_{packet}$ is given by:

(19) $$T_{packet}=\frac{L_{PHY}+L_{MHR}+L_{payload}+L_{MFR}}{R_{data}}$$

where $L_{PHY}$ is the length of the PHY header in bytes, $L_{MHR}$ is the length of the MAC header in bytes, $L_{payload}$ is the length of the data bytes in the data packet, $L_{MFR}$ is the length of the MAC footer in bytes, and $R_{data}$ is the data transmission rate.

## 5. Performance Evaluation

In this section, the performance of the proposed PA-MAC is evaluated via computer simulation and compared with the IEEE standard 802.15.4 and the conventional priority-based MAC. The four performance metrics-the average transmission time, the network throughput, the average energy consumption, and the collision ratio are evaluated.

### 5.1. Simulation Environment

The performance of the proposed PA-MAC is evaluated and compared with the IEEE 802.15.4 standard using an ns-2 Network simulator, Version 2.35. The ns-2 simulator is a discrete event simulator targeted at networking research and provides substantial support for the simulation of various network protocols over wired and wireless networks [28]. 20% of the total nodes generate emergency traffic; the on-demand traffic and the non-medical traffic categories each constitute 20% of the total nodes and the normal traffic occupies 40% of the total traffic generated during each simulation. The physical layer parameters are defined according to the IEEE 802.15.4 standard. We have assumed that the several biomedical sensors are implanted or attached to the human body. The star topology, in which the central coordinator is the master node, is used in our simulation as in other research works. The sensor nodes are randomly deployed within an area of 4 m radius, around the central coordinator and the data are transmitted by one hop. All the nodes intend to transmit the first packet randomly during the contention access period. Small-scale fading has been neglected and it is assumed that packet loss is solely because of collision. The Poisson arrival is used to approximate the random packet arrival process. For medical traffic, a payload size of 40 bytes is used owing to lower end-to-end latency and an acceptable packet delivery rate [11,27]. Emergency traffic occurs randomly and the packet size is the same as the normal medical traffic. The network parameters used in the simulation are summarized in Table 2.

### 5.2. Simulation Results and Discussion

**Figure 10:**
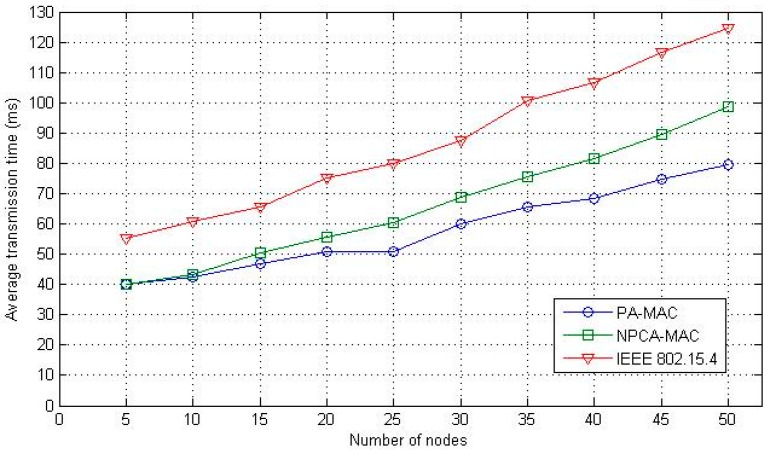
Average transmission time.

The overall performance of the average transmission time is illustrated in Figure 10. In the proposed MAC, a fixed dedicated channel is assigned for the beacon. The WBAN utilizes the single channel statically; the channel access opportunities experience less interference and interruptions. Moreover, the proposed PA-MAC and the conventional NPCA-MAC perform slotted CSMA/CA with a priority-based channel access policy, whereas, the IEEE 802.15.4 MAC protocol operates the slotted CSMA/CA without a priority-based channel access policy. Thus, as in Figure 10, the overall average transmission time of the IEEE 802.15.4 protocol has the largest delay, compared to the proposed PA-MAC and the NPCA-MAC. Additionally, the PA-MAC exhibits a better performance than the NPCA-MAC, as the number of nodes increase.

**Figure 11:**
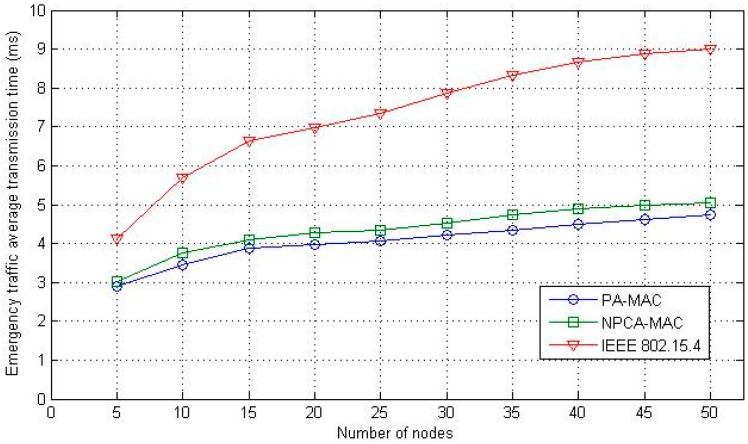
Emergency traffic average transmission time.

In the NPCA-MAC, continuous and discontinuous data transfer procedures and the use of GTSs are not considered. There is no difference in the transmission of emergency traffic in the proposed MAC and the NPCA-MAC. Figure 11 shows the emergency traffic average transmission time for the proposed PA-MAC, NPCA-MAC, and the IEEE 802.15.4 MAC. The main contributor to the transmission delay is the channel access delay. The emergency nodes have to transmit a small-size data packet in a very small time interval. If the channel becomes extremely busy, the sensor nodes have to back off more periods to complete the channel access, thereby causing longer channel access delays. Here, we can see that the average transmission time of all the three protocols increases with the increase in the number of sensor nodes. However, the proposed PA-MAC and the NPCA-MAC exhibit a better performance than the IEEE 802.15.4 MAC protocol.

**Figure 12:**
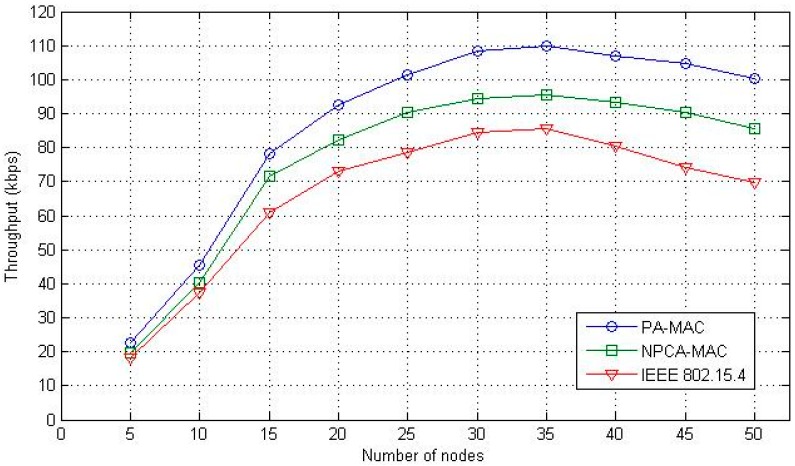
Network throughput.

In Figure 12, the overall performance of the network throughput as a function of the number of nodes is illustrated. All three schemes show a similar performance, when the number of sensor nodes is less than 10. The proposed PA-MAC and the conventional NPCA-MAC, however, provide better performances compared to the IEEE 802.15.4 MAC protocol. In the IEEE 802.15.4 MAC, the collision ratio increases sharply with the number of sensor nodes. Hence, more resources are wasted on data packet collision rather than on effective data transmission. The throughput of all the three schemes seems to decrease as the number of nodes exceeds 35, because of a high contention complexity and an increased packet collision rate. Although the collision rate increases with the number of data packets, the radio resource on the data channels is efficiently managed in both the PA-MAC and the NPCA-MAC, according to the data traffic. However, in the channel access pattern, prioritization of data traffic, and the GTS allocation for continuous data traffic, the proposed PA-MAC performs better than the conventional NPCA-MAC.

Energy efficiency is a key parameter in the design of efficient and reliable MAC protocols for the WBAN. Energy consumption is related to node behavior. A network with busy traffic has a higher energy consumption compared to a network with less traffic. To evaluate the energy efficiency comprehensively, the average energy consumption per bit is used. The average energy per bit is given by:

(20) $$E_{b}=\frac{E_{avg}}{S_{b}}$$

where $E_{avg}$ is the average energy consumption and $S_{b}$ is the throughput achieved.

**Figure 13:**
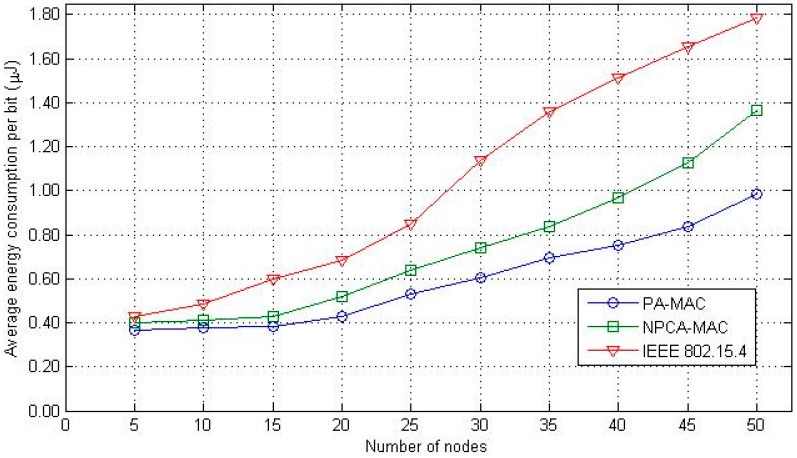
Average energy consumption per bit.

The evaluation of the average energy consumption per bit is shown in Figure 13. The increase in energy consumption is mainly because of packet collision and packet retransmission. The energy consumption of the IEEE 802.15.4 MAC protocol increases sharply with the number of nodes, because of high contention complexity. High contention complexity causes a high packet collision rate and results in a large number of retransmissions. The traffic prioritization scheme reduces the contention complexity and decreases packet collision and packet retransmission. The proposed PA-MAC and the conventional NPCA-MAC show better performances than that of the IEEE 802.15.4 MAC protocol. Above all, the proposed PA-MAC prioritizes channel access and incorporates the classification of the data transfer procedure, thereby reducing the contention complexity, the packet collision, and the packet retransmission. Therefore, the proposed PA-MAC outperforms the conventional NPCA-MAC and the IEEE 802.15.4.

**Figure 14:**
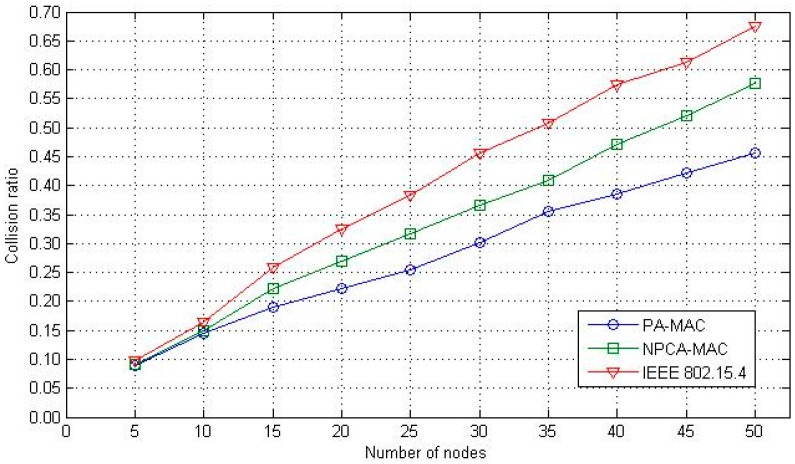
Collision ratio.

Figure 14 shows the collision ratio of the overall traffic in a network as a function of the number of nodes. The number of collisions increases with the increase in the number of sensor nodes in the WBAN. In the IEEE 802.15.4, a slotted CSMA/CA without a prioritization policy did not solve the contention complexity problems; the collision ratio increased discernibly when the number of nodes exceeded 20. The proposed PA-MAC and the NPCA-MAC provide a low collision ratio as compared to the IEEE 802.15.4 MAC protocol, owing to the prioritization of data traffic and the classification of continuous and discontinuous data transfer procedures. With features including channel access patterns and GTS allocations for continuous data traffic, the proposed PA-MAC outperforms the conventional NPCA-MAC.

## 6. Conclusions

Sharing of the ISM band leads to unpredictable service interruptions because of mutual interference between coexisting systems. WBANs operating in highly coexistent interferences may be affected by beacon drops, data collisions, packet delays, low network throughput, and high-energy consumption. To address these issues, in this paper, a priority-based adaptive MAC protocol called the PA-MAC, has been proposed for WBANs in unlicensed bands. A fixed dedicated channel is assigned for the beacon and the rest of the communication is through the data channel. We have also differentiated the access phase of the CAP and have classified the transfer procedure of the priority-based traffic in WBANs. The proposed PA-MAC supports both CAP and CFP. The CFP is used to transfer continuous and large numbers of data packets to the coordinator. According to the simulation results, the PA-MAC shows substantial improvements in terms of transmission time, throughput, energy efficiency, and collision ratio, compared to the IEEE standard 802.15.4 and the conventional NPCA-MAC.

In the proposed MAC, it is assumed that the coordinator and sensor nodes are within the communication range and their mobility is not critical for communication between the coordinator and sensors. Any security and privacy mechanisms are not considered, either. In the proposed MAC, the GTS slots are assigned for both medical data traffic and CE traffic, but the number of GTS slots is limited and, thus, the substantial high collision ratio might result in significant performance degradation, especially in case of heavy and high data rate traffic. Functional extension of the PA-MAC for WBANs with a cognitive ratio capability may be undertaken in the future.

## Figures and Tables

**Table 1:** Different levels of traffic priority.

Traffic Category	Priority	Example
Emergency traffic	*P*_1_ (highest)	Emergency alarm signal
On-demand traffic	*P*_2_	Continuous medical signal (e.g., EEG, EMG)
Normal traffic	*P*_3_	Discontinuous medical signal (e.g., temperature, blood pressure)
Non-medical traffic	*P*_4_ (lowest)	Audio/Video/Data

**Table 2:** Simulation Parameters.

Parameter	Value
Channel rate	250 kbps
Frequency band	2.4 GHz
Symbol time	16 µs
Superframe duration	122.88 ms
Transition time	192 µs
aUnitBackoffPeriod	20 symbols
macMaxCSMABackoffs	5
macMinBE	3
macMaxBE	5
Idle power	712 µW
Transmission power	36.5 mW
Reception power	41.4 mW
